# The rapidly changing field of predictive biomarkers of non-small cell lung cancer

**DOI:** 10.3389/pore.2024.1611733

**Published:** 2024-06-17

**Authors:** László József Tóth, Attila Mokánszki, Gábor Méhes

**Affiliations:** Department of Pathology, Faculty of Medicine, University of Debrecen, Debrecen, Hungary

**Keywords:** NSCLC, driver oncogenes, immune checkpoint inhibitor, gene fusion, biomarker

## Abstract

Lung cancer is a leading cause of cancer-related death worldwide in both men and women, however mortality in the US and EU are recently declining in parallel with the gradual cut of smoking prevalence. Consequently, the relative frequency of adenocarcinoma increased while that of squamous and small cell carcinomas declined. During the last two decades a plethora of targeted drug therapies have appeared for the treatment of metastasizing non-small cell lung carcinomas (NSCLC). Personalized oncology aims to precisely match patients to treatments with the highest potential of success. Extensive research is done to introduce biomarkers which can predict the effectiveness of a specific targeted therapeutic approach. The EGFR signaling pathway includes several sufficient targets for the treatment of human cancers including NSCLC. Lung adenocarcinoma may harbor both activating and resistance mutations of the EGFR gene, and further, mutations of KRAS and BRAF oncogenes. Less frequent but targetable genetic alterations include ALK, ROS1, RET gene rearrangements, and various alterations of MET proto-oncogene. In addition, the importance of anti-tumor immunity and of tumor microenvironment has become evident recently. Accumulation of mutations generally trigger tumor specific immune defense, but immune protection may be upregulated as an aggressive feature. The blockade of immune checkpoints results in potential reactivation of tumor cell killing and induces significant tumor regression in various tumor types, such as lung carcinoma. Therapeutic responses to anti PD1-PD-L1 treatment may correlate with the expression of PD-L1 by tumor cells. Due to the wide range of diagnostic and predictive features in lung cancer a plenty of tests are required from a single small biopsy or cytology specimen, which is challenged by major issues of sample quantity and quality. Thus, the efficacy of biomarker testing should be warranted by standardized policy and optimal material usage. In this review we aim to discuss major targeted therapy-related biomarkers in NSCLC and testing possibilities comprehensively.

## Introduction

Primary lung cancer used to be a rare tumor in the past, however today it is the most common cause of cancer mortality worldwide. In 2020, lung carcinoma was the second most common malignancy, with around 2.2 million newly diagnosed cases. Moreover, with 1.796 million cases, it was the number one cause of cancer deaths [1]. The incidence in both of US and EU is now declining, that follows the trend of shrinking smoking prevalence [2]. The incidence rate in male patients increased from 1973 to 1984 (83.5 and 97.9/100,000 person-years, respectively) followed by a gradual decrease till 2015 (55.3/100,000 person-years), while in female patients the incidence increased in a more extended period from 1973 to 2007 (20.2–51.3/100,000 person-years) and then subsequently decreased to 2015 (44.2/100,000 person-years). The trend in the incidence of lung carcinoma in men in Central Europe is similar to that seen in Western countries, but with a slight delay. Unfortunately, according to the latest data, the incidence of lung cancer is still rising in women [3].

About 85% of lung cancer cases are non-small cell carcinoma, the remaining 15% belong to the clinically separate category of small cell carcinoma. The group of non-small cell carcinomas is further subdivided as adenocarcinoma, squamous cell carcinoma, large cell carcinoma, and several other relatively rare histological types [4]. The relative frequency of histological types of lung carcinoma has also changed significantly in recent decades. In the past the squamous cell carcinoma was the most common type of lung cancer but their relative frequency declined and the general occurrence of adenocarcinoma increased. Today, adenocarcinoma is the most common type, accounting for about 50%–56% of all lung cancer cases and is the most frequent histological subtype in never-smokers [5]. Increased risk of adenocarcinoma in smokers is a result of changes in design and composition of tobacco products. The introduction of ventillated filters in cigarettes and the increased levels of tobacco-specific nitrosamines both have played a role [6–10]. The decline of squamous cell carcinoma follows the trend of declining smoking prevalence in industrialized countries [2].

As incidence and mortality data indicate, lung cancer is one of the most aggressive cancers and has an unfavorable outcome. Classical treatment options for lung cancer include surgical resection or chemo- and radiotherapy, depending on clinic-pathological variables. For patients with early-stage lung cancer, surgical resection is the optimal treatment option, while patients with locally advanced or metastatic NSCLC and most SCLC patients are treated with chemo-radiotherapy. However, in the last decades, significant progress has been made in understanding the molecular pathogenesis of lung tumours, both NSCLC and SCLC groups. Even SCLC, which was previously thought to be uniform, has been shown by recent data to be divided into at least 4 distinct molecular subgroups [11].

Thanks to the massive increase in genetic and immunological knowledge the variety of treatment methods has also shifted over the decades. Therapeutic targeting of the EGF-receptor introduced the era of biological therapies, and a growing list of specifically acting agents is now effectively used in selected cancer patients. So-called oncogene-addicted NSCLC is a molecular genetically distinct group of lung cancers in which well-defined driver mutations direct the pathogenesis, and pharmacological blockade of this target is expected to result in a significant therapeutic response. These patients also form a clinically well-defined group, mostly non-smokers, female predominance, and a relatively younger age are the main characteristics.

**FIGURE 1:**
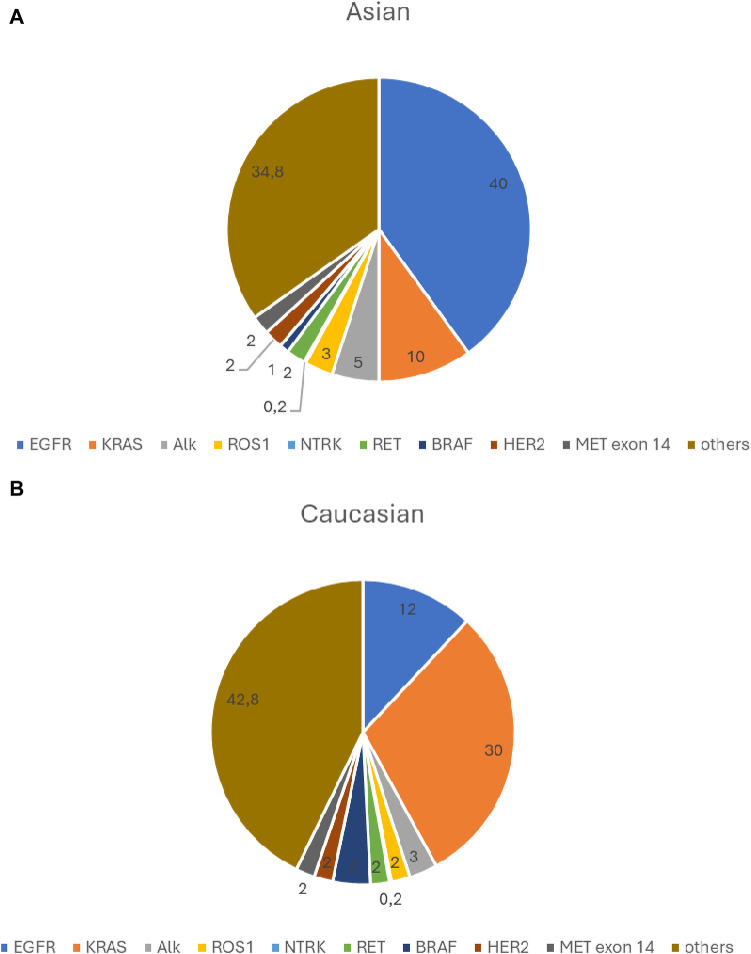
**(A)** Significant driver gene defects frequency- Asian population. **(B)** Significant driver gene defects frequency- Caucasian population.

The frequency of currently known clinically significant driver gene defects is shown in Figure 1.

In addition, another group of lung tumours is also emerging, lacking targetable driver mutations to our present knowledge. However, due to marked immunogenicity, these patients may well benefit from immune checkpoint inhibitor therapies [12].

The introduction of molecularly targeted therapies promise a major advance, however lung cancer still has a poor prognosis and the 5-year survival rate remains at a very low level, the 5-year OS was 10.7 in 1973 for all lung cancer patients, which increased to 19.8% in 2010 [13, 14]. As seen in Western countries, survival rates for lung cancer patients in Central Europe have improved over the past decade, particularly after the introduction of immunotherapy. For non-squamous NSCLC, the 3-year survival in 2019 was 28.7% compared to 14.5% in 2011, and for squamous cell carcinoma 22.3% versus 13.37%. Unfortunately, for SCLC, there was no significant improvement over the study period [15].

The emergence of new molecular targets has also challenged diagnostic pathology by requiring the identification of appropriate predictive biomarkers and by the development of reliable, cost-effective testing options.

In this study, we aim to review the main events in the molecular pathogenesis of NSCLC also serving as therapeutic targets. Furthermore, we aim to present the status of biomarker testing options.

## Gene mutations and copy number changes

### EGFR mutations

Adenocarcinomas harbor mutations of genes of EGFR signaling pathway. EGFR belongs to the epidermal growth factor RTK (receptor tyrosine kinase) family. The EGF receptor has an extracellular ligand-binding, a transmembrane and an intracellular domain, the latter having tyrosine kinase activity. The intracellular protein kinase domain contains a small N-terminal lobe and a larger C-terminal lobe. The two parts form the active site cleft that serves as a binding site for ATP. In physiological conditions the extracellular ligand binding (EGF) activates the receptor and the downstream signaling results in cellular transactivation, cell proliferation and survival.

The use of the EGFR receptor as a potential therapeutic target was already suggested in the 1980s by Mendelshon and colleagues [16, 17]. As in many other tumors, EGFR expression has been shown to be increased in lung adenocarcinomas, an early event in carcinogenesis [18]. In 2002, the first data on an EGFR inhibitor treatment in NSCLC were published [19, 20]. The first clinical results showed considerable variability in the response rate [21, 22]. Benefits were mostly observed in non-smokers, women and in the Far Eastern population. The level of EGFR expression detected by immunohistochemistry has not been shown to be of predictive value [23, 24]. Mutations in EGFR were first identified in 2004 and have also been associated with response to therapy [25–27]. All the detected genetic abnormalities were heterozygous, and wild-type EGFR was found in normal lung tissue adjacent to the tumor, suggesting that these mutations are somatic [25]. The EGFR mutation could also be detected in the benign epithelium surrounding the tumor, demonstrating that it is an early event in carcinogenesis [28]. Mutations were demonstrated to affect the kinase domain of the receptor protein causing constitutive activation and downstream signaling in the absence of the receptor ligand. Women and never-smokers were preferentially involved. EGFR mutations show ethnic differences, prevalence ranges 10%–15% in Caucasians and 30%–40% in Asians [29]. Genetic alterations of other major oncogenes, such as KRAS, ALK, ROS1 are mutually exclusive with EGFR mutations. Among adenocarcinomas EGFR mutations are frequently detected in cases with lepidic and papillary growth patterns. The two most common mutations, the so called classical EGFR mutations are i) mutation at codon 858, replacing leucine 858 with arginine (L858R) and ii) the in-frame deletion in exon 19 causing removal of amino-acid residues 746–750 of the expressed protein [30]. These two mutations account for 85%–90% of all EGFR mutations.

Both classical EGFR mutations affect the kinase domain. In the inactive form of the wild-type EGFR molecule an outward rotation of the alphaC helix in the N lobe is provided, which is stabilized by the helical turn of the A-loop. This conformation inhibits the association of amino acids K745 with E762 and the consecutive binding and orientation of ATP. The L858R mutation occurs in the C lobe N-terminal portion of the activation loop resulting the destabilization of the inactive state thereby promoting the conversion in a more active state [31, 32]. The deletion in exon 19 (746ELREA750) occurs immediately before the αC-helix in the N lobe and desrupts the inactive conformation through the shortening of the alpha C loop. The L858R mutant is approximately 50-fold more active than the wild-type enzyme, and the G719S mutant shows about ten times more activity over the wild-type.

In addition to the classic mutations mentioned above (L858R and del19), about 600 other rare EGFR mutations have been described, accounting for about 10%–15% of all EGFR mutations [33]. These include exon 18 E709x, del18, G719x, exon19 insertion, exon20 insertion, and S768I, as well as L861Q affecting exon21. Of these, exon 20 insertions are the most common (4%–10% of all EGFR mutations).

**FIGURE 2:**
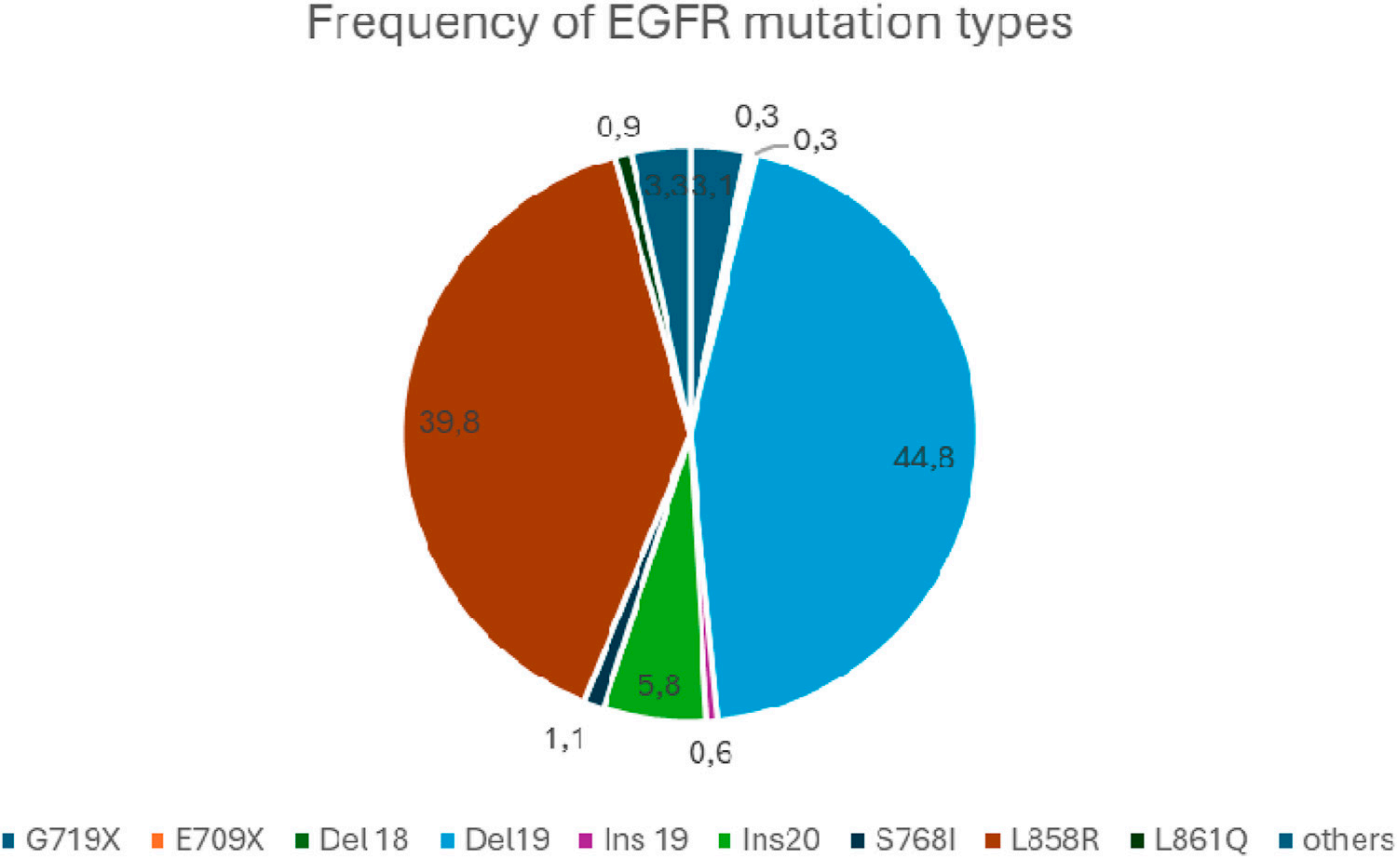
The relative frequency of EGFR mutations.

The relative frequency of EGFR mutations based on the COSMIC database is shown in Figure 2 [34].

EGFR mutations show variable sensitivity to EGFR tyrosine kinase inhibition. The specific genotype obtained by DNA sequencing gains special importance as the type of mutation carries the basic potential of resistance to given inhibitor agents. It is of note that resistance or low response to TKI today indicates the consideration of an alternative inhibitor. No response to EGFR TKI treatment has been observed in case of exon 20 insertions [35] except A763-Y764insFQEA [36–38]. For others, the therapeutic effect is less than observed for the classical mutations, e.g., G719x, exon 19 insertions, S768I, L861Q. For these mutations 2nd generation TKI treatment may show improved results.

Germline mutations of the EGFR gene were also described [39], which, if present, increase the incidence of adenocarcinoma, mostly in the presence of additional somatic mutations. The 4 most common germline mutations are T790M (which is one of the most important causes of acquired TKI resistance), V843I, R776H, P848L, all point mutations.

An EGFR activating mutation is a prognostic factor indicating unfavorable outcome. On the other hand, it is a factor predicting the response to tyrosin kinase inhibitor treatment. T790M mutations and mutations in exon 20 are associated with resistance to tyrosin kinase inhibitors.

Although EGFR-TKI treatment has been shown to be effective in most patients with EGFR mutant lung adenocarcinomas, no response is observed in 5%–25% of patients due to intrinsic resistance to these drugs. In addition to the pharmacokinetic complications of the drug, intrinsic resistance is frequently due to some genetic variation. These include most of the exon20 insertions, with the exception of A763-Y764insFQEA- [36–38], T790M, exon 2-7 variant III(vIII) in frame deletion [40] and some other secondary genetic events.

**FIGURE 3:**
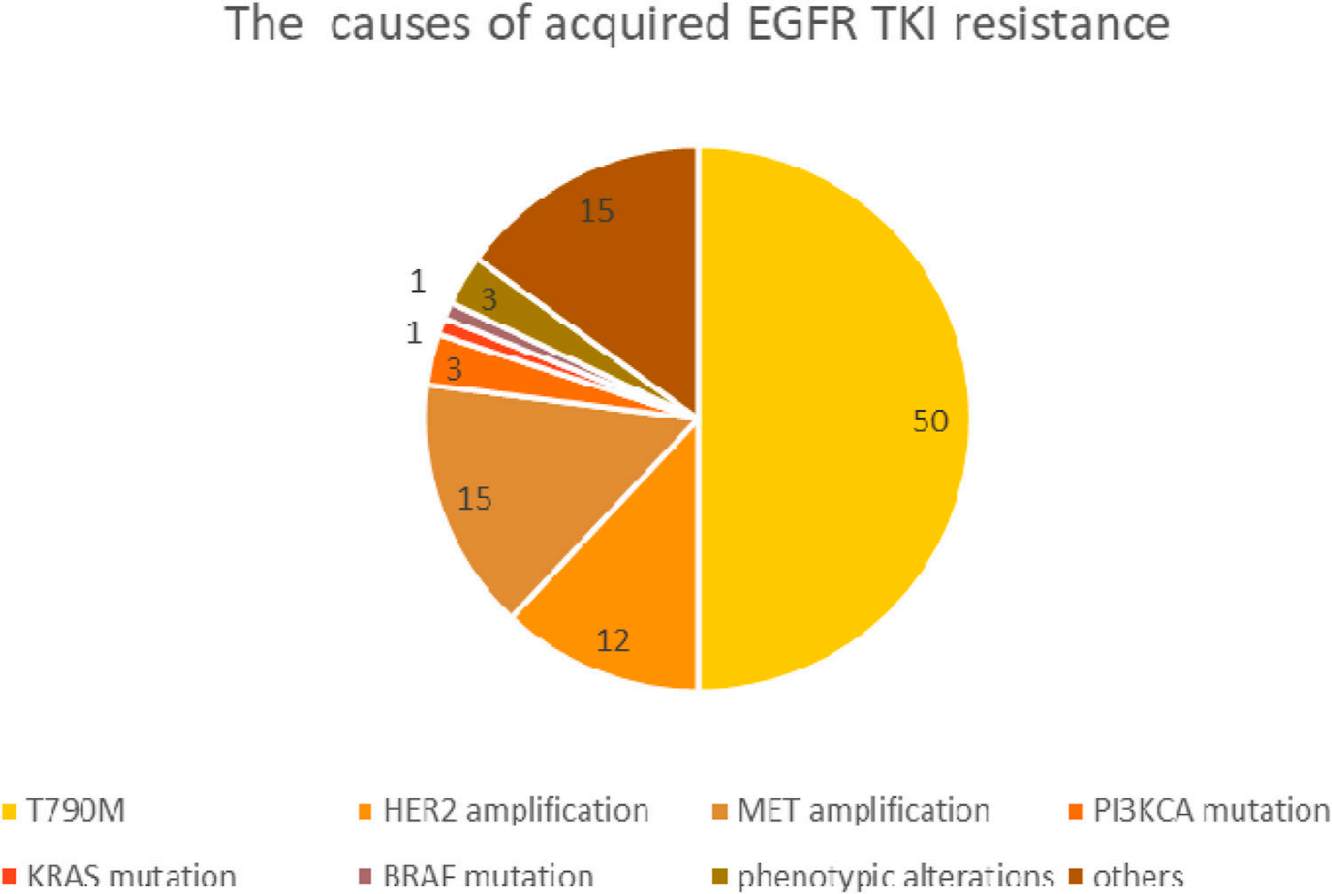
The most common causes of acquired EGFR TKI resistance.

Patients with an initially good therapeutic response may show unfortunate disease progression after 9–19 months of treatment initialization due to acquired resistance (first line TKI: 9–12 months, 19 months first line third generation TKI) [41, 42]. Acquired resistance is often the result of a secondary EGFR mutation, while in many cases it occurs due to activation of an alternative signaling pathway. The most common (49%–63%) acquired mutation is T790M, which results in a threonine-methionine substitution at position 790, the ATP binding site of the receptor protein. This amino acid exchange prevents EGFR-TKI from binding to the kinase domain and results in increased ATP affinity. Further to *de novo* mutagenesis it is assumed, that a co-existing, drug-resistant EGFR T790M positive subclone has been selected by TKI treatment. In addition to the T790M mutation, EGFR gene amplification and several rare second/third EGFR mutations are considered as resistance mechanisms, including the C797S, L792, L718Q, SV768IL genotypes. The activation of alternative signaling pathways may also occur through a gene amplification, such as MET, HER2 or other rare gene mutations. Resistance may also result from tumor phenotypic alterations, such as transformation towards squamous or small cell carcinoma and epithelial-mesenchymal transition [43, 44]. The most common causes of acquired EGFR TKI resistance and their relative frequency are shown in Figure 3.

It is surprising that, despite the wealth of data that has been accumulated on the incidence and therapeutic significance of EGFR mutations, very little is known about the origin and causes of EGFR mutations. Few publications correlate different etiological factors and EGFR mutations. It appears that air pollution, including exposure to microparticles, can induce EGFR mutations [45]. In addition, exposure to radon in the residential environment may also have a potential EGFR mutation-inducing effect [46].

EGFR genotyping is generally based on sequence analysis of tumor DNA isolated from biopsy samples. As a fast alternative approach to predict the efficacy of EGFR inhibitory treatment, the expression of total EGFR protein was first attempted for immunohistochemistry, but as already mentioned, this remained inconclusive [23, 47] Mutation-specific monoclonal antibodies against exon 19 E746-A750del and exon 21 L858R mutations are available, which represent approx. 90% of all EGFR mutations (clone 6B6 for exon19 deletions; and clone 43B2 against point mutation of exon21 L858R, Cell Signaling Technology) [48]. Tests with these antibodies have shown relatively high sensitivity and specificity for the two mutations indicated, especially for L858R. The lower sensitivity for exon19 deletions is mostly explained by the fact that the exon19 antibody only detects the most common deletion of exon19 (deletion E746-A750). This deletion is of 15 base pairs and represents 50%–65% of all exon19 deletions. However, a variety of deletions, including 9, 12, 16, 18, and 24base-pairs variants have been identified, each producing slightly different protein and antigen epitope structure not detectable by the commercial exon19 antibody [49]. According to the literature the EGFR mutation-specific antibodies have a fair sensitivity and high specificity in identifying lung adenocarcinomas with classic EGFR mutations, while they do not recognise uncommon EGFR mutations. They did not provide sufficient sensitivity (about 40%–60%) or specificity (70%) for the detection of all EGFR mutations, compared to gold standard sequencing methods [50–53]. Sanger DNA sequencing has been widely used, but its disadvantages, primarily its low sensitivity (requirement of 40%–50% mutant DNA in samples) [54], has before led to the development of more sensitive detecting methods including real-time quantitative PCR (RT-PCR). However, this method is relatively expensive, time consuming, and not incorporated in routine diagnostic procedures in many departments of pathology. In contrast, immunohistochemistry has lower costs, shorter turnaround time, and is available in the majority of laboratories. IHC is a rapid, cheap and well-known assay that does not require huge tumor cell content and performs quite well even in degraded tissue (e.g., decalcified bone tissue) or cytology samples. EGFR-mutant-specific antibodies cannot replace conventional molecular methodologies, but they could be very helpful in small tumor samples with poor material [53].

### KRAS mutations

KRAS is one of the longest known oncogenes and activating mutations play a crucial role in the early oncogenesis of several types of tumors, such as pancreatic ductal adenocarcinoma, colon cancer and lung cancer [55]. HRAS and NRAS are the other two members of the RAS gene family with clinical impact [56]. The human homologue of the RAS gene, HRAS, located on the short arm of chromosome 11 at position 11p15.1-11p15.3, was first described in the early 1980s in a human bladder cancer cell line. A short time later KRAS, a gene showing homologue features was detected in lung adenocarcinoma, located on the short arm of chromosome 12 at position 12p11.1-12p12.1. The NRAS gene is located on chromosome 1 [57]. All three RAS genes have 4 exons and broadly similar structures [58]. The KRAS gene encodes two protein isoforms composed of 188 and 189 amino acids (KRAS-4B and 4A), the single amino acid difference is a result of alternative splicing [59]. The KRAS protein is a cell membrane-associated G protein with GTP-ase activity [60,61], coupling cell surface growth factor receptors such as EGFR to various intracellular (mitogenic) signaling pathways. The most common signaling pathways involved are the mitogen activated protein kinase (MAPK) [62, 63] and phosphatidylinositol 3-kinase (PI3K) pathways [64]. The active RAS protein binds GTP, in which guanine nucleotide exchange factors (GEFs) participate through GTP-GDP exchange. GTPase activating proteins (GAPs) inactivate RAS by enhancing the GTPase activity of the RAS [60, 61, 65]. RAS gene mutations at specific sites result in spontaneous activation of the RAS protein without mitogen signaling, leading to uncontrolled cell proliferation and cell survival. As these play an important role from the earliest phase of carcinogenesis they are called oncogenic, or hot-spot mutations [55, 66] In the case of KRAS G12C or G12V mutations, intracellular levels of RAS-related proteins (RALs) are elevated and AKT phosphorylation is reduced [67].

The earliest and most frequent mutations in lung adenocarcinoma occurs in the KRAS gene and affects the EGFR/RAS/RAF signaling pathway [68–70]. The prevalence of the KRAS mutation is around 30% in the Western population, compared to around 10% in the Far East. In lung cancer, mutations in NRAS or HRAS are rare, with a prevalence of less than 1% each. The vast majority of KRAS mutations (about 80%) affect codon 12 within exon 2 of the gene and is close to the section encoding the nucleotide binding site of the KRAS protein. The most common change of codon 12 mutations is a G>T transversion, which results in a glycine-cysteine (G12C) or glycine-valine (G12V) substitution at the protein level. Another type of codon 12 mutation is G>A transition, replacing amino acid glycine by aspartic acid (G12D). Less frequent codon 12 mutations are also known: G12A, G12S, G12R, G12F. Occasionally, mutations can be detected in codon 13 (G13C) or codon 61 (Q61H) [71].

**FIGURE 4:**
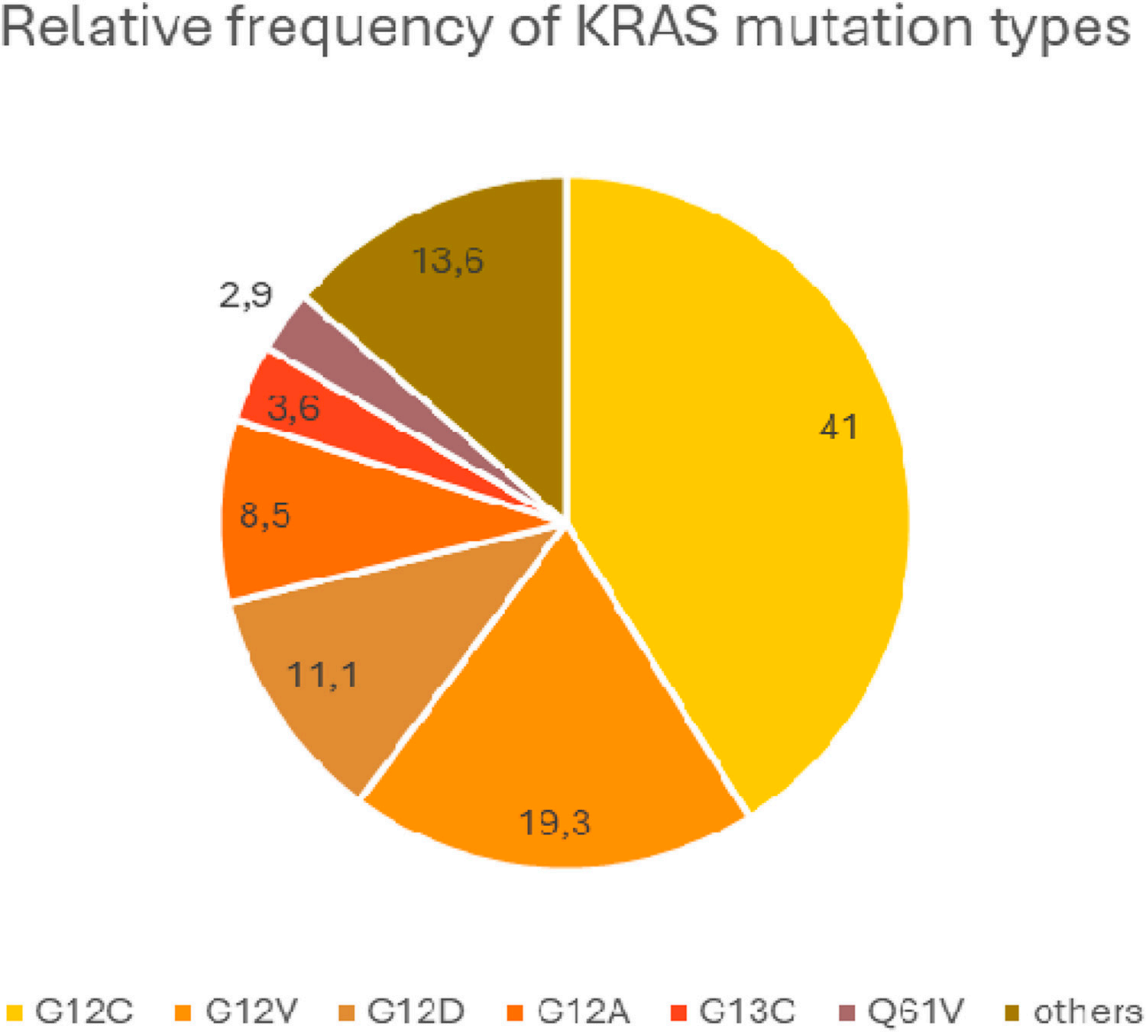
Average relative frequency of the most common KRAS mutation types.

Mutations generally inhibit the interaction of KRAS with GAPs and the hydrolysis of GTP bound by KRAS, thereby keeping the KRAS protein in an active conformation [72]. Different mutations activate different intracellular signaling pathways to different degrees. In KRAS G12C mutant tumors, the classical mitogenic signaling pathway (RAS-RAF-MEK-ERK) is activated, whereas the PI3K-AKT-mTOR pathway is dominant for the other mutant genotypes. This may be due to a different RAF affinity in each mutation type [73] The G>T transversions resulting in G12C exchange are supposedly associated with polycyclic aromatic hydrocarbons (PAH) exposure in tobacco smoke. On the other hand, G>A mutations resulting in KRAS G12D are more common in non-smokers. The prevalence of KRAS mutations is 40% in heavy smokers, 30% in former current non-smokers and 20% in never-smokers [74]. The most common type is G12C, which accounts for 40% of all KRAS mutations, while G12V and G12D account for 19% and 11%, respectively. The average relative frequency of KRAS mutation types, based on large databases, is shown in Figure 4.

There are also significant geographical and social differences in the prevalence of KRAS G12C, with a higher prevalence in Western countries and a lower prevalence in the Far East (8.9%–19.5% and 1.4%–3%, respectively), which may be related to smoking habits. Our own observation also suggests this, as KRAS G12C is more common in Eastern Hungary, the care area of our centre, than in the better developed western part of the country, which may be explained by the smoking habits of the population here (unpublished data). Although KRAS mutations, especially G12C, are strongly associated with smoking, KRAS mutations are surprisingly unfrequent in small cell lung cancer, which occurs almost exclusively in heavy smokers [74, 75]. In squamous cell carcinomas, which are also strongly associated with smoking, the occurrence of KRAS mutations is rare or only occurs in mixed tumors, such as adenosquamous carcinoma [71]. The frequency of KRAS mutations also varies in the histological subtypes of adenocarcinoma. KRAS mutations are most often detected with mucinous morphology, but different genotypes show variable frequencies (G12C rarely, whereas G12V and G12D more often) [76]. Among mucinous tumors, KRAS mutations are almost exclusively found in invasive mucinous adenocarcinomas (69%), with no KRAS mutations detected in adenocarcinoma *in situ* and colloid carcinoma. However, mucinous carcinomas do not harbour EGFR mutations [77]. Interestingly, the KRAS mutation is more common in women [78]. The metastatic pattern of the tumor also differs with the mutant genotype, with G12C tumors having a higher incidence of intrapulmonary metastasis (38% vs. 21%) and a lower incidence of pleural metastasis (4% vs. 39%). It was also observed that brain metastasis is less frequent in KRAS mutations (33% vs. 40%), but the frequency of brain metastasis is similar for each KRAS mutation type [79, 80].

The presence of EGFR and KRAS mutations are mutually exclusive. In theory they affect the same signaling pathway and the presence of one mutation is sufficient for tumourigenesis. Despite this generally accepted view, KRAS and EGFR mutations can rarely occur within the same tumor [76, 81]. A recent large retrospective study of data from 3,774 patients found the concomitant presence of two or three driver mutations in 1.7% of cases, most commonly EGFR/KRAS mutations (in 0.53% of cases). It should be noted that this study examined a patient population from the Far East, where the prevalence of EGFR mutations is inherently high, 43% in this study [82].

The particular role of KRAS in tumorigenesis is also suggested by the observation that in one of the most common congenital pulmonary malformations, congenital pulmonary airway malformation type I (CPAM), KRAS mutations (G12V or G12D) are often detected, predominantly in intracystic mucinous cell clusters. In fact, these mutations can be identified in nonmucinous cystic regions as well, but not in healthy lung tissue. In these patients, KRAS mutant adenocarcinoma of mucinous character frequently appears later, indicating to the oncogenic nature of the mutation [83–85].

The prognostic role of KRAS mutations in lung adenocarcinoma has long been the subject of intensive studies. Although many data have been published on this issue, its prognostic significance is still not clearly understood. Investigations of prognostic significance are complicated by the fact that the term “KRAS mutation” itself is not uniform, mutation types, ethnic, gender and histological differences as well as treatment mode should also be considered. In addition, other genetic abnormalities, such as EGFR mutations, modify the behavior of KRAS wild-type cases and affect prognosis [86]. However, most of the large case-control studies published in recent years support the hypothesis that the presence of KRAS mutations, particularly KRAS G12C mutations, has a negative prognostic significance, and reduce both OS and PFS [87–90]. There is also evidence that increased mutated KRAS levels in circulating tumour DNA also have a negative prognostic significance [91].

The predictive significance of the KRAS mutation has also been extensively studied. Many reports suggest that mutant KRAS is not a negative predictor of conventional chemotherapy [92] However, as with prognostic significance, the question is more nuanced, with many factors to consider, such as the type of KRAS mutation, the patient population studied and treatment characteristics. In early-stage resected NSCLC patients, no significant predictive value of KRAS mutation status was found for adjuvant treatment [93], and similar results were obtained in the neoadjuvant setting [94]. A more recent study showed that KRAS mutation is a negative predictor of cytotoxic chemotherapy in advanced NSCLC [95].

Another important question is the impact of KRAS mutations on targeted therapies, especially EGFR targeting. Many conflicting observations have been reported on this topic, too. Most studies have shown that the presence of a KRAS mutation has a negative predictive effect in this respect, EGFR TKI treatment in these patients having a worse objective response rate. However, no difference in survival has been found between KRAS mutant/EGFR wild-type and KRAS wild-type/EGFR wild-type patients, therefore, the significance of KRAS mutation to select patients for EGFR TKI treatment appeared to be limited [96]. The results of individual studies are significantly affected by the type of KRAS mutation present: while poor treatment efficacy is seen with G12C and G12V, better response rates are seen with G12D and G12S [97].

Testing for KRAS mutations is possible from tumor DNA isolated from tumor tissue, bronchial brush smears, plasma or pleural fluid (cfDNA analysis), using either a single-gene PCR-based approaches, classical or a next-generation sequencing [98, 99].

### BRAF mutations

BRAF is a member of the rapidly accelerated fibrosarcoma (RAF) kinase family. Its role is signal transduction from the RAS protein to the mitogen-activated protein kinase cascade (MAPK) [100]. The RAF protein is composed of three main domains: CR1, CR2, CR3. CR1 functions as an auto-inhibitor of the kinase CR3 domain and is also responsible for RAS-GTP binding. The CR2 region forms a flexible link between the CR1 and CR3 domains. Upon activation the RAS-GTP binds to the RAS-GTP binding site (RBD) of the CR1 domain. BRAF is then phosphorylated on amino acids T599 and S602, which results in a protein conformational change. This active form homo- or heterodimerizes with other RAF family proteins, also contributing to the stabilization of the active conformation. The results is the activated BRAF kinase domain, which phosphorylates MEK1, the downstream member of the MAPK signaling pathway [101].

BRAF is one of the most frequently mutated genes in human tumors. Mutations are most frequently detected in melanoma (40%–50% of cases), but are also common in papillary thyroid cancer, colorectal cancer and NSCLC [102–105].

(It is interesting to note that Davies [102] was the first to report the BRAF mutation, but in her publication she uses a different nomenclature, V599E, to refer to the mutation he detected - now called V600E-because the sequence of the protein had previously been misinterpreted, A31 G32 A33 was mistaken for R31 P32. Because A33 was missing from earlier sequences, some studies incorrectly assigned wrong numbers to coding mutations and amino acids.)

BRAF mutations most commonly occur at codon 600 in exon 15 of the gene, resulting in the exchange of amino acid valine to glutamate (V600E) of the protein. Other substitutions, such as V600D/K/R, can be also rarely seen at this site. These mutations are also known as class I mutations [106]. The V600E mutation results in a marked increase in BRAF kinase activity (up to 500-700-fold compared to wild-type BRAF), with a consequent stimulation of the MAPK signaling pathway. The V600E mutation results in the conformational change of the monomer BRAF protein already possessing with an active kinase function that would otherwise gain through dimerization of the wild-type conformation [107]. In melanoma, V600E is the most common BRAF mutation, whereas in NSCLC only half of the mutations affecting BRAF belong to this group. NonV600E mutations form a heterogeneous group and can be further classified into class II and class III [101].

BRAF mutations play an important role in lung carcinogenesis, as also demonstrated experimentally *in vivo* [108]. Data from large case-control studies suggest that BRAF mutations occur in 2.2%–4.9% of NSCLC. Owsley found 772 BRAF mutations (4.1%) out of 18,944 NSCLC cases, of which 30.7% (237 cases) were V600E mutations [103]. In Villaruz’s study, 21 cases (2.2%) of 951 adenocarcinomas proved to be BRAF mutant, 81% of which were V600E [109]. V600E mutations occur mainly in women, non-smokers, are associated with micropapillary morphology and have a worse prognosis than wild type [110]. Non-V600E mutations are exclusively detected in smokers, in equal proportions in men and women, and are not associated with a prognosis worse than the wild type (Marchetti, 36 BRAF mutations detected in 739 adenocarcinomas- 4.9%- of which 56.8% were V600E) [111]. The results of individual studies are contradictory regarding the prognostic role. Villaruz et al. observed a better outcome for V600E, while others did not observe any difference in prognosis [109, 112] The contradictory results are likely explained by the fact that BRAF mutations are relatively rare and only small cohorts of patients could be examined despite extended studies. Co-mutations are relatively common, mainly mutations in KRAS and PI3K, while in nonV600E, mutations in p53 and STK11 are common [106].

The conformational change through the mutation enables the differentiation of the BRAF protein from the wild-type form. Thus, the BRAF status can also be assessed by immunohistochemistry for V600E mutations. The commercially available monoclonal diagnostic antibody was raised against a synthetic version of the V600E-encoded protein fragment located around the amino acid affected by the mutation [113]. This antibody detects the BRAF V600E mutant epitope with sufficient sensitivity and specificity, as has been demonstrated in several tumor types such as colorectal carcinoma, papillary thyroid cancer and melanoma [114–118] However, it is not applicable to V600D/K/R or class II and III non-V600E mutations. Since almost half of the BRAF mutations in NSCLC are nonV600E, the IHC test is of limited use to identify tumors harbouring BRAF mutations. However, according to current NCCN recommendations, specific TKI inhibitor treatment should be used for V600E mutation positive tumors [119]. Thus, the IHC methodology may be considered as a screening test for the identification of these patients in histological conditions. In addition, the same guidelines (and updated version also) recommend an NGS-based methodology to determine a comprehensive BRAF status [120].

## Gene fusions with clinical relevance

In addition to the now “classic” MAP-kinase pathway mutations, several clinically relevant chromosomal rearrangements have also been identified in NSCLC. It has long been known that specific gene fusions determine the development of several haematological malignancies and sarcomas. The earliest such gene fusion identified was bcr-abl characteristic for chronic myeloid leukaemia, discovery finally leading to the pioneering concept of tyrosine kinase inhibitor therapies [121]. Oncogenic gene translocations play a special role in NSCLC carcinogenesis, especially if their functionality can be therapeutically blocked. The most important ones are ALK, ROS1 and RET rearrangements and the significantly less frequent NTRK gene fusions, with prevalence rates of 4%–6%, 2%, 1%–2% and 0.1%–0.23%, respectively. These fusions occur in a patient population clinically distinct from classical NSCLC (predominantly younger, non-demented patients with adenocarcinoma histology).

Due to the availability of effective targeted TKI drugs with FDA or EMA approval it is particularly important to identify these patients within the confines of predictive molecular testing. The selection of sufficiently effective and validated, yet rapid and not least relatively inexpensive methodology is a major challenge for pathology laboratories and molecular geneticists. In addition to the “big four,” additional gene fusions have recently become known, such as those involving NRG1, SMARCA4, BRAF, FGFR1 and EGFR, further complicating the everyday molecular diagnostic practice of NSCLC.

### ALK rearrangements

The ALK gene (anaplastic lymphoma kinase) was discovered as a result of genetic studies in anaplastic large cell lymphoma. The gene is located on the short arm of chromosome 2, in the 2p23 region. As a member of the insulin receptor superfamily, ALK encodes a tyrosine kinase-activated transmembrane receptor protein whose function is only partially understood. In humans, ALK expression is detected intermittently during neural development, with a decline in expression during postnatal life. In adults, it is expressed only scattered in some neurons, endothelium and in pericytes of the brain. The ALK protein contains an extracellular ligand-binding, a transmembrane, and a cytoplasmic kinase domain [122]. The ligand(s) for the ALK receptor have not been unequivocally identified. The role of pleiotrophin, midkine was hypothesized [123], followed by other candidates, including heparin, FAM150A and FAM150B [124].

The main types of ALK gene alterations include rearrangements (fusions), gene amplification, and point mutations [125].

Fusions of the ALK gene, like the amplification of the gene leads to constitutive activation. Amplification of ALK has been detected in neuroblastoma [126] breast cancer, anaplastic large cell lymphoma and pulmonary sarcomatoid carcinoma [127]. On the contrary, point mutations are the most common causes of resistance to ALK TKI treatment. Known resistance mutations are G1269A, C1156Y, L1196M and several other point mutations [128, 129].

The 2p chromosomal region is sensitive to genotoxic effects, favoring the breakage of the ALK gene, with the result of gene fusions and increased expression of the kinase domain of the ALK protein. In 1994, a t [2, 5] translocation was first described in anaplastic large cell lymphoma, resulting in an NPM (nucleophosmin)-ALK fusion gene, an event that is detected in 60%–80% of ALCL [130]. It has subsequently been described in additional tumors: e.g., inflammatory myofibroblastic tumor [131], colorectal and breast cancer, and esophageal squamous cell carcinoma [132]. Further fusion partners were later identified. The setup of the rearrangements is common in that the breakpoint leaves the entire ALK tyrosine kinase domain intact, while the promoter region always comes from the fusion partner. The fusion partner also contains an oligomerization domain, the presence of which allows ligand-independent constitutive activation of the receptor protein.

In 2007, Soda and colleagues detected the EML4 (echinoderm microtubule associated protein-like 4)-ALK gene rearrangement in NSCLC. This gene rearrangement is caused by an inversion of the chromosome region 2p21-23. The extracellular and transmembrane regions of ALK are replaced by EML4. There are various EML4 breakpoints and therefore, several variants of the fusion gene are known [133]. The EML4-ALK gene rearrangement results in constitutive activation of ALK RTK, an oncogenic pathway in NSCLC. The resulting EML4-ALK fusion gene product represents a novel molecular target for the treatment of non-small cell lung cancer. ALK gene rearrangement occurs in 3%–6% of all NSCLC. It is typically associated with adenocarcinoma morphology (including papillary, mucinous, and squamous cell variants) [134]. Detected mainly in non-smokers or light smokers and typically in young patients. ALK gene rearrangement, EGFR and KRAS mutation are mutually exclusive events [135].

The demonstration of EML4-ALK gene rearrangements was challenging due different variants of the fusion, requiring multiplex testing in the PCR era. FISH-based detection could be adopted with satisfying efficacy since the 3′and 5′ends of the ALK gene get separated due to the rearrangement, and their labelling with separate fluorescent probes result in the characteristic split signal. Currently, the FISH test is the gold standard for the detection of ALK rearrangements in clinical samples, requiring specific probes, fluorescence equipment and properly experienced pathologist.

Because of the above drawbacks, an immunohistochemical alternative for the detection of ALK gene involvement has been attempted. This assumes that ALK protein is not expressed in normal lung tissue, but gene fusion and ALK gene activation result in moderately increased expression of ALK protein. However, the detection of the protein underwent an evolution. A “traditional” diagnostic antibody (ALK1) previously used for anaplastic lymphoma was not sufficiently sensitive (sensitivity 67%, specificity 97%). However, the release of new antibody clones promptly followed [5A4, D5F3, anti-ALK (1A4)] and the use of highly sensitive amplification systems allowed to achieve adequate sensitivity and specificity. The advantage of IHC testing is its low cost, wide availability and rapid turnaround time. In immunohistochemistry, the ALK fusion protein shows granular cytoplasmic staining. In signet ring cells (a morphology often seen in ALK-positive adenocarcinoma), staining is present along the membrane in a thin rim that can be difficult to distinguish from background staining. Several studies have demonstrated that the use of properly validated antibody and immunohistochemical platform, together with an external control (e.g., appendix with intense ALK positivity in the ganglion cells of the wall), provides a highly reproducible result. The study of Mino-Kenudson et al. in 2010 (n = 153, sensitivity 100%, specificity 99.0%) using clone D5F3 [136] and that of Paik et al. in 2011 (n = 735, sensitivity 100%, specificity 96.2%) using clone 5A4 both showed high concordance between Ventana IHC and FISH results [137].

The high sensitivity of IHC to detect ALK aberrations is today generally accepted. Despite the rare IHC negative but FISH positive cases published in the literature, the current recommendations accept the use of IHC methodology without FISH confirmatory testing in histological specimens [138] as well as in cell block specimens prepared from malignant pleural effusions [139]. However, the IHC methodology is neither perfectly applicable nor validated in large series on bronchoscopic brush cytology specimens. For cytology preparations the FISH break apart test should primarily be chosen.

### ROS1 rearrangements

The ROS1 gene is located on chromosome 6 in the 6q22 region. The gene was originally discovered in the 1980s during the study of avian sarcoma viruses. The human ROS gene is homologous to the v-ros proto-oncogene of the UR2 sarcoma virus [140, 141]. The protein contains an extracellular and an intracellular domain, the latter having tyrosine kinase activity with structural similarity to the ALK protein. The physiological role of the ROS1 gene and protein is poorly understood, but it is thought to be involved in differentiation signaling pathways of various epithelial tissues during embryonic development [142]. ROS1 protein expression is observed in the kidney, the cerebellum, the peripheral nerves, various parts of the alimentary canal, but is not expressed in lung tissue under normal conditions [143]. The physiological ligand of the receptor is still debated. The structure of the extracellular domain suggests that cell adhesion plays a role in its activation [142] The intracellular signaling pathways activated by ROS1 are also not well understood, but induction of MAPK and PI3K pathways is hypothesized [144]. Its oncogenic relevance was first demonstrated in the early 2000s in glioblastoma [145]. In lung tumors, rearrangements affecting ROS1 gene were first described in 2007 [146]. ROS1 involvement was observed in 1%–2% of NSCLC [147]. As a result of the rearrangement, various breakpoints in the ROS1 gene may evolve, including exons 32, 34-36, or introns 31 or 33. The fusion gene commonly contains the tyrosine kinase domain of ROS. There are 9 different fusion partners known in lung cancer, such as FIG, CD74, SLC34A2 and SDC4, EZR and the list is growing [148, 149]. The oncogenic mechanism of gene rearrangement is not understood. ROS1 rearrangements typically occur in lung adenocarcinomas, rarely in adenosquamous carcinoma. These adenocarcinomas generally show a solid pattern, frequently of signet ring cell type. Younger patients and non-smokers are more frequently affected [150]. It is often detected at an advanced stage and brain metastasis is common [151]. Interestingly, lung carcinoma patients with ROS1 gene rearrangements have a higher incidence of paraneoplastic thromboembolic events [152].

Due to the larger set of fusion variants the ROS1 rearrangement can be most effectively demonstrated by FISH analysis, using ROS1 specific DNA-probes and the detection strategy of the ALK testing. Sequencing by different NGS platforms is also applicable if tumor tissue and appropriate amounts of DNA or RNA are provided. Rearrangement of the gene is associated with overexpression of the ROS-protein, allowing the use of ROS1-specific antibodies. For IHC-based diagnostic testing, the use of the D4D6 ROS1 antibody clones is recommended. In positive cases, fine granular cytoplasmic staining is observed. Fusion variants show a different staining pattern, which may be the result of the intracellular function and localization of the fusion partner. For CD74-ROS1, a globular pattern with randomly arranged cytoplasmic granules of 6–8 mm diameter and weaker background cytoplasmic staining was described, explained by the fact that CD74 is associated with intracellular membrane systems. Membranous staining was seen in the presence of EZR-ROS1 fusion, presumably due to the ezrin protein binding to plasma membrane and actin cytoskeleton [153] A uniform scoring system for the ROS1 IHC reaction is still missing, most studies use the H-score calculated from the staining intensity and the proportion of positive tumor cells. With appropriate preanalytical and analytical standards, this antibody can achieve high sensitivity (95%–100%) but relatively poor specificity (63%–90%) [154–157]. The low specificity may potentially originate from a moderate ROS1 expression by macrophages, reactive alveolar epithelium, or even by tumors without ROS rearrangement. A higher cut-off value results in a higher specificity. Overall, the IHC test has a high negative predictive value and is therefore suitable as a screening test, with a negative IHC result virtually ruling out the presence of ROS1 fusion. A positive IHC result requires confirmation by genetic means, such as FISH or NGS technology [158, 159]. Since ROS1 rearrangements are rare, the relatively simple IHC staining is highly effective and avoids the mass need for expensive molecular testing [138, 160].

### RET rearrangements

The RET oncogene was identified in the 1980s by transfection of DNA extracted from a human T-cell lymphoma cell line [161]. The RET gene is located on chromosome 10 at position 10q11.22 [162] and encodes a transmembrane tyrosine kinase growth factor receptor. Its extracellular domain contains 4 cadherin-like structures [163]. Its ligand is glial cell line-derived neurotrophic factor [164, 165]. Ligand binding results in dimerization and activation of the receptor, which then activates several intracellular signaling pathways such as PI3K/AKT, RAS/RAF/MEK/ERK or JAK2/STAT3. RET activity is important for kidney and nervous system development, gene expression is precisely regulated in space and time during embryogenesis [165]. RET is required for the proper development of the enteric nervous system, in particular for the migration of neural crest cells and enteric neurons into the wall of the alimentary canal. The absence of RET expression or activity is associated with the development of Hirschsprung disease (segmental aganglionosis of the colon) [166].

Various genetic defects within the RET gene are also related with carcinogenesis. CCDC6-RET fusions have been detected in thyroid cancer as early as in 1990 [167]. Further to oncogenic fusions, activating point mutations of the RET gene are also known. They are involved in the development of medullary thyroid carcinoma and MEN2A syndrome, among others [168, 169]. In NSCLC, RET gene rearrangement was first reported in a Korean non-smoking male patient in 2012 [170–173]. Several large studies have reported that RET rearrangement is present in 1%–2% of NSCLC (Takeuchi 0.9% in 1482 NSCLC, Qiu 1.4% in 1587 NSCLC) [149, 173]. Following the summary of data from 4857 NSCLC patients from previous studies, the prevalence of RET rearrangement proved to be 1.4%, while in the adenocarcinoma group of 3,576 patients 1.8% had RET fusions [174]. KIF5B-RET fusion was observed with the highest prevalence (52%), this type was typical for women, while CCDC6-RET was mostly observed in men with a prevalence of 26% in total. In addition, several rare fusion partners have been described (MIR392, ZBTB41, ITGA8, SLC39A8 [149]. Along with the RET rearrangements, several other genetic events may be detected; [175]. It is known that RET fusions are responsible for acquired therapy resistance during EGFR TKI inhibitor treatment in 1%–2% of the cases, mainly involving CCDC6 as the fusion partner; [176]. In most RET rearrangements, the transmembrane domain is lost, resulting in a chimeric cytosolic protein that exerts its oncogenic effect through constitutive activation of the kinase domain [161] The majority of RET rearrangements, like ALK and ROS1 fusions, occur in young, non-smoker or mild smoker women with lung adenocarcinoma diagnosis; [173, 177]. This patient group has a significantly higher incidence of brain metastases both at diagnosis (27%) and overall during the course of the disease than the RET wild-type group [178].

The demonstration of RET gene fusions in clinical samples is of great importance, as there are several FDA-approved small-molecule inhibitors promising effective treatment. Initially, non-specific multikinase inhibitors were used, more recently followed by RET-selective inhibitors [179–182].

RET rearrangements can be demonstrated by several alternative methodologies. Although immunohistochemistry is a widely available method and has proven useful in detecting ALK and ROS1 rearrangements, its value in detecting RET fusions is unfortunately limited. The IHC methodology using RET specific antibodies had low sensitivity and specificity, with a false positive rate of 62% and a false negative rate of 46%, in other words, RET rearranged samples could not be equivocally identified. These results have been unanimously confirmed by several studies, and therefore the use of IHC is not recommended for the diagnosis of RET gene rearrangement [183, 184].

On the contrary, the FISH methodology has been shown to be successful in detecting a substantial amount of gene rearrangements. Using RET gene region specific probes FISH had a high sensitivity of 100% for the chimeric proteins KIF5B and CCDC6 but less than 100% for the other partners. On the other hand, a surprisingly poor specificity of 45%–60% was measured, therefore, the currently available DNA probes have not been recommended for routine diagnostics of RET gene fusions [184, 185]. Considering all these issues NGS remains the optimal tool for general RET testing. DNA-based NGS showed a sensitivity of 87.2%–100% for detecting RET fusions, while its specificity was also highly satisfying (98.1%–100%) [186].

### NTRK fusions

In humans, three neurotrophic tyrosine receptor kinase (NTRK) genes are known, encoding the transmembrane neurotrophin receptors TrkA, TrkB and TrkC. These Trk receptors are involved in embryonic development of the central and peripheral nervous system [187]. In adults, they are expressed only in neural tissue and skeletal muscle [188]. Ligand-dependent activation of Trk receptors activate several biochemical pathways, including MAPK and PI3K signaling [189]. Chromosomal rearrangements of NTRK genes result in increased expression and/or activation of Trk receptors [190]. The occurrence of NTRK gene fusions is characteristic for some rare tumor types, such as mammary analogue secretory carcinoma of the salivary gland or congenital infantile fibrosarcoma. ETV6-NTRK3 rearrangements are detected in 90% of these cases [191, 192]. Although NTRK gene rearrangements are generally rare, they have been detected in a broad range of common solid tumor types. NSCLC, colorectal carcinoma, GIST, papillary thyroid carcinoma, melanoma, pancreatic adenocarcinoma and gliomas were reported with very rare NTRK involvement of less than 1% of cases [193]. In NSCLC, the incidence rate was only 0.1%–0.3%, an order of magnitude lower than the frequency of ALK or ROS1 gene rearrangements [194]. In two very large NSCLC case-control studies, the rates of NTrk fusions were 0.1% (Gatalica, 4,073 lung adenocarcinomas [195]) and 0.23% (Solomon, 3,993 lung adenocarcinomas [196]). NTRK1 fusion could be demonstrated most (68%), followed by NTRK3 (24%) and NTRK3 fusion as the least common change. Because of the rarity of occurrence, even the largest studies had limitations defining detailed clinical characteristics of patients with NTRK fusions. They suggest equal distribution in both women and men, with a wide age range. The majority of those carrying the fusion are non-smokers, but heavy smokers were not excluded. Most NTRK-positive tumors proved to be adenocarcinomas with mucinous or poorly differentiated morphology, but fusions have also been detected in neuroendocrine carcinoma and even squamous cell carcinoma [197].

Although rare, the identifications of these tumors opens the way for NTRK-targeted TKI therapy, that is available in the last couple of years promising a favorable therapeutic response in patients with NTRK gene fusion [198, 199]. In a 2023 study, 51 patients with advanced NSCLC harboring an NTRK fusion had an ORR of 62.7% following entrectinib treatment, while the PFS and OS was 28.0 and 41.5 months, respectively [200]. Predictive testing of NTRK gene fusion-due to the altogether 3 TrK genes and numerous fusion partner genes-is quite cumbersome, even by the classic FISH arrangement. Due to the 3 independent NTRK gene regions potentially involved, three FISH assays and tests would be required. However, while the detection of fusions involving the NTRK3 gene by FISH had good sensitivity, too many false negative cases were reported for NTRK1 fusion detection. This may be due to intrachromosomal rearrangements involving a short segment, allowing only limited signal separation in the break-apart probe assay, causing interpretation difficulties. Another problem appeared as FISH probes could not detect rearrangements with some fusion partners. Because of these drawbacks, NTRK FISH were not recommended for routine diagnostics [201]. As an alternative, RNA-based massively parallel sequencing (MPS) is considered as a favorable methodology. However, this technique is not widely available, not mentioning the turnaround time and costs of the test.

Taking everything together, immunohistochemical detection of Trk proteins as a screening test should be considered. Immunohistochemistry is available in virtually all pathology departments, is relatively rapid and inexpensive and sufficiently works with small amounts of tumor tissue. Commercially available diagnostic antibodies detect all Trk proteins. The staining pattern is variable: membrane, cytoplasmic and nuclear positivity can all be present. Unfortunately, a validated scoring system is not available at this time. A positive tumor is defined as one with at least 1% of tumor cell positivity, any kind of positivity more intense than background should be satisfactory, regardless of the staining pattern. Confirmatory testing of positive cases by MPS seems to be necessary for proper interpretation.

According to the literature the sensitivity of the IHC method ranges from 75% to 97% and the specificity is remarkably high, reaching 98%–100% [202–204]. Previous large studies have shown that the sensitivity of IHC is not uniform for the three NTRK gene fusions, 96.2%, 100% and 79.4% sensitivity rates were measured for NTRK1, -2, and -3, respectively, while the specificity was 81.1%. More specifically for the lung adenocarcinoma patient group, IHC sensitivity was 87.5% and specificity 100% [196]. The above large studies all used the Abcam EPR17341 antibody clone. However, in a more recent study, 133 (14.8%) out of 1068 NSCLC cases were panTrk IHC positive, but only 2 cases could be confirmed by RNA-based testing, resulting a positive predictive value of 1.5% for the IHC test applied [205]. Unlike in previous studies, in this work the C17F1 antibody clone was used and any staining was accepted as positive. In conclusion, predictive IHC testing of NTRK involvement also should be performed with care. Sensitivity and specificity rates may be strongly influenced by the type of the diagnostic antibody used. However, sensitivity of IHC supposed to be relatively lower for NTRK3 fusions, the reason of which is not known in detail. If uncovered, low sensitivity of the IHC screening could drop out patients of an effective treatment opportunity.

## Novel driver gene defects in NSCLC

### MET alterations. Met exon 14 skipping mutations

The mesenchymal epithelial transition (MET) proto-oncogene is located in t4.1he chromosomal region 7q21-q31 and encodes a transmembrane receptor tyrosine kinase protein [206, 207]. The MET protein is expressed in diverse cells of epithelial origin, and is further expressed in liver cells, endothelial cells and neurons. The ligand for this receptor is hepatocyte growth factor (HGF), which is mainly produced by mesenchymal cells, such as fibroblasts [208]. The extracellular part of the receptor protein is responsible for ligand binding and includes domains like the semaphoring and the immunoglobulin-plexin transcription factor domain. The intracellular part is composed of the juxtamembrane domain and the catalytic domain [209, 210]. Ligand binding activates the protein by causing homodimerization, which then leads to autophosphorylation of tyrosine residues in the catalytic domain. Activated MET induces several intracellular activation pathways through MAPK, PI3K, Nf-kB and signal transducer and activator of transcription3 (STAT) signaling. HGF/MET activation plays a key role in epithelial-to-mesenchymal transitions (EMT) by regulating extracellular matrix adhesion and cytoskeletal changes [211, 212]. The deactivation mechanism of the activated signaling pathway deserves attention, as changes in this process play a key role in the carcinogenic effect of MET [213]. After ligand binding, homodimerisation and intracellular signaling the active receptor protein is internalized by clathrin-mediated endocytosis, it is partially degraded but recycling and return to the cell membrane is possible. This process is controlled by ubiquitin ligase casitas B-lineage lymphoma (CBL), which recognizes the Tyr1003 residue encoded in exon 14 of the MET gene and the ubiquitinated MET is degraded by the endosome system [214, 215].

Genetic events may affect MET protein function resulting in oncogenic effects. MET gene amplification results in increased expression and constitutive activation of the kinase protein. This mechanism is supposed to be responsible for acquired resistance during EGFR TKI treatment in 3%–4% of [42, 216, 217]. Various point mutations have also been detected in several tumours, including lung carcinoma, but their oncogenic role remains unclear [218]. Moreover, some gene fusions have also been described, such as KIF5B-MET, which have potential oncogenic effects and serving as therapeutic targets [219].

Exon 14 mutations are the best known MET alterations with pathogenetic and apparently, clinical significance. This specific mutation type is a result of a two base pair insertion in intron 13. The insertion represents an alternative mRNA splicing site with the consequence of the “skipping” of the entire exon 14 during translation for protein synthesis. Therefore, the functional molecule lacks the juxtamembrane segment containing the Tyr1003 residue responsible for the internalisation of the activated receptor. Thus, the exon 14 skipping mutation enhances the stability of activated MET on the surface of the cell, resulting in a prolonged activity of the receptor signaling [220, 221]. According to one of the first large case-control studies, this mutation is present in about 3% (131/4,402) of NSCLC cases [222]. According to a recent large meta-analysis, exon14 skipping mutations can be detected at a rate of 2% in NSCLC, no significant geographical differences are reported. The prevalence proved to be 12% in non-smokers and 2% in smokers, with a similar overall prevalence of MET14 skipping mutation positive patients with a history of smoking and non-smoking. It is more common in women and at older age (average is 73 years). It is noteworthy that while the histological type of adenocarcinoma has a prevalence of 2.4%, it is detected in 12% of sarcomatoid carcinomas [223]. Association with major driver mutations (EGFR, KRAS, BRAF and ALK, ROS1 gene fusions) appears to be rare, but some other genetic events, such as MET, EGFR amplification, or PI3K mutations may co-occur [222]. Data to date suggest that MET exon 14 mutations in NSCLC are associated with a poor prognosis.

The therapeutic targeting of MET kinase is relevant with multiple TKI drugs (e.g., tepotinib, capmatinib, savolitinib) resulting in good therapeutic response [224, 225]. This is true for the exon-skipping alteration but also for the Y1003N mutation of the juxtamembrane domain, which also inhibits CBL-mediated degradation [222].

Due to the heterogeneity of Met exon 14 aberrations, their detection is another challenge for diagnostic pathology laboratories. Attempts have also been made to detect MET alterations by immunohistochemistry. While overexpression of MET protein can be detected in many tumor types in many cases, MET overexpression can be detected in 35%–72% of NSCLC by immunohistochemistry [226, 227]. Unfortunately, in the majority of IHC positive cases, there is no Met exon 14 skipping or MET amplification. As shown in a recent study, 71 tumors in 181 NSCLC cases had MET overexpression detectable, but only 1% of IHC positive cases had amplification and 3% had Met exon 14 skipping. These results also show that IHC-based detection is not a suitable screening test for MET alterations [228]. RNA-based assays have the highest sensitivity and can detect exon 14 skipping independent of the underlying diverse genetic alterations by detecting fusions of exons 13 and 15 following mRNA transcription. The disadvantage of the RNA-based methodology is its sensitivity to RNA degradation [229]. Unfortunately, amplicon-based DNA NGS tests have a detection rate of only 63% [230], whereas hybrid-capture NGS methodology can achieve better results, but requires larger amounts of sample DNA, which is frequently not provided from small biopsy samples [231]. To overcome this issue circulating free DNA (liquid biopsy) should have lower sensitivity but a high positive predictive value [232].

### HER2 alterations

HER2 (ERBB2) is a member of the HER growth factor receptor family. This family also includes EGFR, EGFR3 and EGFR4. The HER2 gene is located in the 17q11.2-q12 region. The encoded receptor protein has an extracellular ligand-binding, a transmembrane and an intracellular tyrosine kinase domain, like other growth factor receptors of the family [233]. It is unique compared to other members of the HER family in that it has no known ligand but has an intrinsic tyrosine kinase activity and is specifically prone to homo- or heterodimerization, which results in its activation. A frequent heterodimerization partner is HER3. Activated HER2 can activate several intracellular signaling pathways such as MAPK, PI3K, and STAT [234].

Several alterations may occur in the HER2 gene, which have oncogenic effects. Gene amplification of HER2 is well known in breast cancer, one of the oldest known therapeutic targets [235], but is also common in gastric [236] and ovarian cancer. HER2 alterations can also occur in lung carcinoma, but gene amplification is relatively rare. However, HER2 amplification in NSCLC may be a potential cause of acquired resistance during EGFR TKI treatment [42]. HER2 amplification is most easily detected by FISH, which is analogous to the common testing practice in breast carcinoma, with a HER2/CEP17 hybridization signal ratio greater than 2. Importantly, HER2 overexpression is often detected by immunohistochemistry, which is usually due to a balanced increase of copy number (polysomy). In this case the HER2/CEP17 ratio does not exceed 2 determined the FISH analysis [237]. Various mutations in the HER2 gene may also occur in the coding regions of all three domains with a rate of 2%–4% of NSCLC. In the first study, published in 2004, HER2 mutations were presented in 10% of the lung adenocarcinomas [238] Subsequently, several studies have reported higher case numbers, with lower frequencies (1.6% testing 671 NSCLC cases) [239]. The most common types of mutations proved to be in-frame insertions in the kinase domain coding region in exon 20. These mutations change the protein conformation and increase kinase activity, thereby activating intracellular signaling [240]. HER2 exon 20 insertions are like exon 20 insertions detected in the EGFR gene [241]. Many of these insertions have been described, the most common being the YVMA insertion, which was detected in 68% of the 98 HER2 mutant tumors detected in a study of altogether 2,788 patients [242]. HER2 mutations occur mainly in women, non-smokers, are associated with adenocarcinoma histology and brain metastasis is common in these patients [243]. Another large study reported HER2 mutations in 24 of 920 patients (3%), 71% non-smokers, 58% women, mean age was 62 years [244]. The co-occurrence of HER2 mutations with other classical driver mutations is virtually excluded [239, 245]. However, some HER2 mutations develop in about 1% of cases of acquired resistance to EGFR TKI treatment [246]. Neither immunohistochemistry nor FISH is suitable for HER2 mutation detection. Since many types of these mutations are known, NGS sequencing methodology is the only effective way of testing [247].

## Immune-checkpoint alterations

Modulation of the anti-tumor immune response as another option of anti-tumor therapy becoming part of everyday oncological care. Tumor-related antigens can be recognised by immune cells through the complex process of antigen-presentation and T-cell activation. Ideally, T-cells migrate to the tumor, where they recognise and destroy tumor cells, the efficacy of which is regulated by several receptors and ligands triggering co-stimulatory and inhibitory signals (immune checkpoints). Tumour neoantigens play an important role in the activation of the immune response enabling the separation of tumor from normal cells. The accumulation of mutations in genomically instable cancers is associated with the generation of neo-antigens showing marked immunogenicity. More specifically, the mutational burden is higher in cancers associated with prolonged exposure to environmental carcinogens. Examples include UV radiation in melanoma development, and respiratory carcinogens, primarily polycyclic aromatic compounds in the tobacco smoke, in relation with lung cancer, both small cell and non-small cell type. Increased mutation frequency is a result of insufficient DNA repair mechanisms, e.g., the loss of mismatch repair (MMR) gene functions [248].

The immune checkpoint regulation can be modified by tumor cells, an important immune resistance mechanism. Programmed cell death protein 1 (PD1) is a cell surface immune checkpoint receptor in activated T- and B-lymphocytes, with inhibitory impact on effector cell functions [249]. The PD1 gene is located on chromosome 2 and belongs to the immunoglobulin gene family [250]. The PD1 protein has an extracellular domain consistent with immunoglobulin variable IgV domain, and is featured with an intracellular segment including the immunoreceptor inhibitory tyrosine-based switching motif (ITSM), responsible for inhibition of T-cell activation. The ligand for the PD1 receptor is PD-L1, a cell surface protein containing IgV and IgC domains [251]. PD-L1 can be expressed by T and B cells, macrophages, dendritic cells, and many non-haemopoietic cells. Cells expressing PD-L1 are tolerated by the immune system. Antigen-presenting cells expressing PD-L1 can inhibit T lymphocytes. Thus, the physiological function of PD-L1 is to provide immune-tolerance by inhibiting the adaptive immune response [252]. Unfortunately, tumor cells may also acquire PD-L1 expression in an adaptive or constitutional manner. Adaptive PD-L1 expression occurs in response to interferon-gamma, secreted by T-cells activated by tumor antigens and is mainly observed in the tumor-immune contact zone. Constitutive expression results from activation of various signaling pathways and is uniformly distributed throughout the tumor. Upregulation of PD-L1 ligand by tumor cells inhibits the antitumor immune response in the tumor microenvironment. In many tumor types, including NSCLC, PD-L1 expression is observed in tumor cells, especially in poorly differentiated tumors, and several large meta-analyses have shown that increased PD-L1 expression has a negative prognostic impact [253, 254].

Consequently, the therapeutic blockade of the PD1-PD-L1 relationship represents a promising therapeutic modality in oncology which was outlined by a surprising response in several tumor types from the earliest stage of clinical studies [255]. By now, therapeutic monoclonal antibodies with immune checkpoint inhibitory effect have been introduced for the treatment of a variety of tumor types. However, only about 20% of patients developed an objective response, the rest either having no meaningful effect or developing resistance to treatment. Although ICI treatment does not have serious side effects compared to chemotherapy, characteristic adverse effects may occur, which are often also serious. Therefore, predictive biomarkers for patient selection of PD-L1-PD1 inhibitor treatment would be of particular importance [256]. Unfortunately, there is currently no really good universal predictive biomarker to select patients who could potentially benefit from ICI treatment. In theory, the potential predictive role of factors influencing the tumor-host immune system relationship could be all be considered, including the tumor mutational burden (TMB), the tumor infiltrating lymphocyte (TIL) count, DNA repair systems, in particular mismatch repair and finally the expression of PD-L1. Unfortunately, the predictive clinical value of these factors varies significantly between tumor types.

In NSCLC, the assessment of PD-L1 expression in tumor tissue has been shown to have the strongest predictive value. Early studies have already indicated that the efficacy of PD1 blockade is highly dependent on PD-L1 expression in tumor cells. However, a confusing situation has developed in the field of predictive PD-L1 testing. In a short period of time, four PD1-PD-L1 inhibitor drugs (nivolumab, pembrolizumab, atezolizumab, durvalumab and more recently cemiplimab) have been launched. In parallel, several diagnostic antibodies have been released for the detection of PD-L1 expression. Large clinical studies to test the therapeutic efficacy of their agents applied different antibodies and used different immunohistochemistry platforms. Currently, there are four FDA-approved PD-L1 diagnostic antibodies run on two different IHC platforms: clones 22C3 and 28-8 are determined for the Dako link48 platform (Agilent), and clones SP263 and SP142 for the Ventana (Roche) platform. In addition, various scoring systems for PD-L1 expression have been established. Briefly, the tumor proportion score (TPS) gives the proportion of tumor cells with membrane expression, the combined proportion score (CPS) to assesses the expression of immune cells in the surrounding area in addition to tumor cells, and the IC score to determine the expression of immune cells. Moreover, different cut-off values have been set for the same active substance, depending on whether it is defined for a first-line or a multi-line treatment. For some of the drugs, national medicines authorities insist on the use of companion testing, e.g., the 22C3 antibody Dako link48 for pembrolizumab or SP263 for durvalumab treatment. In other indications the use of PD-L1 testing is complementary, helping to select patients for a more pronounced therapeutic response, thus providing a more accurate assessment of the risk/benefit ratio. Such a complementary test should be used, for example, for atezolizumab with SP142 or for nivolumab with 28-8 antibody clones. There are some indications and drugs where predictive marker testing is not justified under the current pharmacopoeian standards [257]. The results of the KEYNOTE-001 clinical drug trial have shown a strong correlation between the efficacy of pembrolizumab and the level of PD-L1 expression as determined by the 22C3 antibody [256, 258]. The results suggested the utility of 50% TPS as a cut-off, a value which was confirmed by subsequent studies (Keynote-010 and 024). Interestingly, PD-L1 expression as determined by 28-8 antibodies in the Checkmate 017 study was not found to be predictive for nivolumab response. However, the Checkmate 057 study concluded, that PD-L1 expression and clinical response significantly correlate, although only a modest ORR increase was observed as expression increased. Thus, the FDA has accepted PD-L1 detection for this drug as a complementary test [259]. PD-L1 detection for atezolizumab has also been accepted as a complementary test, based on data from the POPLAR and OAK studies [260]. Most recently, cemiplimab has received FDA approval for use, based on the results of the EMPOWER-lung-1 study, and the use of this drug was also linked to greater than 50% PD-L1 expression, determined by SP263 and the Ventana platform as a companion test [261, 262]. The alternatives of specific ICI therapies and PD-L1 predictive testing techniques resulted in differences in the current NCCN and ESMO recommendations, and these are also reflected in the various national medicines regulatory specifications [120, 263].

Taken together the above, a never seen complexity of a biomarker determination can be stated. The variety of different diagnostic PD-L1 antibodies and development platforms and different evaluation systems as well as the growing number of ICI drugs and relevant national recommendations required the comparison of PD-L1 detection methodologies. The results of PD-L1 determination with four commonly available anti-PD-L1 antibodies (22C3, 28-8, SP142 and EIL3N) were compared in a multi-institutional study [264]. In addition to IASCL, the relevant pharmaceutical and diagnostic companies were also involved in the design and conduction of the Blueprint 1 and 2 studies. The very detailed results indicated that the evaluated tests were not always interchangeable. The 22C3, 28-8 and SP263 antibodies and their elicitation systems have been shown to be highly concordant, in terms of sensitivity and specificity, for the determination of TPS. In contrast, SP142 showed a consistently lower TPS value, while 73-10, tested in the Blueprint 2 study, showed a much more intense staining reaction [265, 266].

A major limitation in PD-L1 testing is that it can only be reliably done on embedded tissue samples. Although some studies have reported results of PD-L1 detection on cytological smears (bronchial brush smear, lymph node EBUS guided FNA smear) [267], IHC on this sample type is difficult to standardize and results show a large variability. The large variability of preanalytical characteristics in cytology samples is well known. As indicated in previous studies, fixation is a key pre-analytical factor, e.g., alcohol-based fixatives strongly reduce the feasibility of IHC reactions. Thus, the PD-L1 IHC reaction on smears should be validated in every laboratory. The determination of IC and CPS in cytology is also problematic as the assessment of tumor cell-immune cell relations in direct smears is almost impossible. Larger cell clusters, 3-dimensional clusters, blood contamination hamper the evaluation. In addition, instead of the membranous staining seen in tissue sections, there may be diffuse surface staining on direct smears, mimicking a cytoplasmic reaction [268–270]. As a result, there is a high interpretation and interobserver variability in the assessment of PD-L1 detection when cytology smears are used [268]. For these reasons, the manufacturers of FDA-approved diagnostic antibodies do not recommend the use of cytology smears and users are advised to favor cell blocks. Cytology samples processed in cell block format have been shown to be suitable for PD-L1 detection. This methodology is optimized for and is analytically similar to IHC and allows standardization criteria of the IHC reaction. Several studies have shown satisfactory concordance between the results of PD-L1 detection on cell blocks and tissue samples [271]. In special cases, when the sampling from the tumor tissue (both histology or cytology) fails, it is possible to determine PD-L1 expression from cell blocks of malignant pleural effusion (MPE) samples. Relatively few studies have been performed on this sample type, with small case numbers. The results to date have shown good concordance (85.1%, kappa 0.774) with PD-L1 expression detected in paired primary tumor tissue samples, using three TPS cut-off values. Interestingly, MPE cells appeared to show significantly higher PD-L1 expression (*p* = 0.005) [272]. Our own institute has also had positive experience, successfully using MPE cell blocks in the absence of tissue samples to assess tumor PD-L1 status (data not published) In conclusion, the formalin-fixed paraffin-embedded cell block preparation technology is the ideal alternative to test PD-L1 in cytology specimen [272–274].

The development of pioneering assays to make PD-L1 determination is highly progressive. A new potential tool to assess PD-L1 expression following immunohistochemistry is digital image analysis, with or without the support of artificial intelligence. One such system is the Aitrox AI Model [275]. These digital systems provide powerful assistance in exact quantification and scoring of PD-L1 expression in classic histological conditions.

Determination of soluble PD-1 or PD-L1 in plasma by enzyme-linked immunosorbent assay (ELISA) is another new approach to investigate PD-L1 status. High soluble PD-L1 detected before treatment indicates unfavorable prognosis in ICI-treated lung carcinomas patients, with both PFS and OS being shorter. Changes is sPD-L1 levels after therapy could not be associated with disease outcome. A predictive role of sPD-L1 has not been confirmed to date.

A further promising blood-based assay evaluates exosomal PD-L1 and PD-L1 in circulating tumor cells. In the first series of studies there was no significant correlation between circulating tumor cell PD-L1 expression (neither pre- nor post-treatment) and OS following ICI treatment. However, the dynamic interaction between tumor and immune system was suggested as significantly shorter PFS was observed with high exoPD-L1 levels before ICI treatment, whereas longer PFS was observed with higher exoPD-L1 after treatment.

These plasma-derived PD-L1-associated assays, in addition to simple and non-invasive sampling promise the potential of PD-L1 monitoring to reflect the dynamic, temporal relationship between tumor and immune system. The actual prognostic and predictive role of these biomarkers for ICI treatment is not yet clear [276].

There is generally a negative correlation between tumor PD-L1 expression and response to immunotherapy and the presence of oncogene driver mutations, except for KRAS and partially BRAF and met exon 14 skipping mutations [277]. This correlation has been confirmed in several studies [278, 279]. Since oncogene addicted NSCLC is a rather heterogeneous group both genetically and biologically, there are differences in response to ICI therapy between tumors defined by individual gene defects [280]. Classical EGFR mutations and exon 20 insertions usually show moderate PD-L1 expression and low TMB, and are generally unresponsive to ICI treatment [281]. ALK and ROS1 gene rearrangements often show high PD-L1 expression but low TMB, and these tumours are not responsive to ICI treatment [282, 283]. So strong is this negative correlation that the presence of EGFR and ALK mutations is a treatment exclusion in most ICI recommendations. HER2 mutations are also associated with moderate levels of PD-L1 expression and low TMB detection, and ICI treatments are not effective [284]. RET rearrangements also show low TMB, variable levels of PD-L1 expression and, although there are few and conflicting data, they do not suggest that ICI treatment is effective [285]. The tumours defined by the gene defects listed so far, as previously detailed, are predominantly located in the periphery of the lung and occur mostly in never-smokers or light smokers. Driver mutations play a key role in the formation of these tumours, with escape from immune mechanisms playing a minor role, so that ICI treatment is usually ineffective or results in a modest response [12]. EGFR mutations and driver gene fusions are rare in lung tumors that develop with prolonged exposure to carcinogen tobacco smoke, but KRAS mutations are common [286, 287]. In addition, the tumour mutational burden of these tumours is high. These smoking-associated tumours are markedly immunogenic, and thus tumour formation is influenced by immune escape mechanisms, such as high expression of PD-L1 by tumour cells. Not surprisingly, these smoking-associated tumours with high PD-L1 expression and high TMB generally respond well to ICI treatment [12]. However, due to genetic heterogeneity, there are also significant differences within these tumour groups. In the case of KRAS mutation, good results with ICI treatment are seen in the presence of p53 mutation [288]. Such good results are not observed for STK11 or KEAP co-mutations (KRAS mut/STK11 mut: ORR11.6%, PFS: 2.0 months, OS: 6.2 months, KRAS mut/STK11 wild type: ORR: 32.4%, PFS: 4.8 months, OS: 17.3 months, KRASmut/KEAP mut: ORR: 17.8%, PFS: 1.8 months, OS: 4.8 months, KRAS mut/KEAP wild type: ORR 29.3%, PFS: 4.6 months, OS: 18.4 months) [289]. Among BRAF mutations, a relatively good therapeutic response to ICI treatment is expected in the presence of smoking-associated class II-III non-V600E mutations. In the presence of V600e mutations, only moderate results are observed with ICI treatment [290, 291]. For tumours carrying Met exon 14 mutations, moderate therapeutic response with ICI treatment has been observed [292].

## The everyday challenges of biomarker testing

There has been an explosion of knowledge on the oncogenic mechanisms of NSCLC drivers in recent years. Consequently, molecular biomarkers have become known, which are in use for predictive testing to optimize treatment of patients with advanced lung cancer. The conventional approach to biomarker testing is based on the analysis of tumour tissue samples. The extended needs on different testing platforms require increased amounts of tissue and DNA or RNA extracted. Unfortunately, only about 20% of patients are resectable, and in about 80% of cases the diagnosis is based on small biopsy and/or cytological sample [293]. Previous reports (before 2010) suggested that in up to 70% of all lung tumours, diagnosis was made on cytological specimen alone [294]. In the past decades, the differentiation of SCLC and NSCLC was sufficient, but today the accurate subtyping of NSCLC is required as the effect of targeted treatments is mostly expected in adenocarcinomas [295]. NSCLC subtyping is most reliable when tumor specimens are used but cytology smears are principally useless in specific cases, e.g., PD-L1 determination.

The predictive molecular testing recommendations for lung cancer therapy are constantly changing and in the light of new scientific findings and evolving technologies. Further, there are significant differences between the current NCCN, CAP, ESMO and Pan-Asian NSCLC recommendations [120, 263, 296, 297]. At the beginning of the era only one or two biomarkers needed to be tested, starting with the EGFR mutational status. According to 2023 ESMO recommendations, all advanced non-squamous NSCLC cases should be tested for ALK, ROS1, NTRK, RET fusions, MET exon14 skipping mutations, BRAF, KRAS G12C and HER2 mutations in addition to EGFR mutations. Molecular testing is only justified for squamous cell carcinoma in special circumstances: young age (below 50 years), non-smoker, ex-moderate smoker or long-time non-smoker status. At this complexity the use an NGS testing platform is recommended, if available. Due to the expansion of gene fusions of interest, RNA-based NGS appears to be the best option. In addition, liquid-based cfDNA testing is also acceptable, but in case of negative results, tissue sampling is required [297]. It is reasonable to determine PD-L1 expression by IHC testing for both advanced squamous and non-squamous NSCLC cases [263]. As already detailed above, the available diagnostic platforms and scoring should be carefully applied: testing with 22C3, 28-8, and SP263 antibody clones show a high concordance, whereas the clone SP142 results in a lower TPS [265, 266]. All these factors require a carefully standardized planning of the daily diagnostic practice.

National guidelines based on international recommendations tend to develop, tailored to the national healthcare system and financial resources. Accordingly, there might be significant differences in the national recommendations. While the reflex testing of biopsy specimens from all patients with advanced NSCLC is generally recommended, on-demand testing is preferred in some countries to save costs. Unfortunately, according to a 2018 study, access to molecular testing is more limited in several Central European countries due to limited resources, and in many countries, on-demand testing is preferred to reflex testing [298]. Since the publication of the aforementioned study, there have been several encouraging developments in these countries [299]. The question arises if it is worth to expend resources on quasi-useless testing for patients with poor performance status, ECOG4, who are not suitable for active oncological care. The hierarchy of testing methods should also be considered for the routine detection of rare genetic events. As an example, it is cost-effective to screen for rare NTRK rearrangements by immunohistochemistry and then to confirm only positive cases by sequencing. The most commonly used diagnostic antibodies currently commercially available are summarised in Table 1.

**TABLE 1:** Most common diagnostic antibodies used to test for driver gene alterations or PD-L1 expression.

Driver gene alteration	Antibody clone	Vendor
EGFR L858R [48–52]	43B2 SP125	Cell Signaling Ventana
EGFR del 19 (E746-A750) [48–52]	6B6 SP111	Cell Signaling Ventana
Alk [136, 137]	D5F3 5A4	Cell Signaling Novocastra
ROS1 [153, 155]	D4D6	Cell Signaling
RET [185]	EPR2871	Abcam
NTRK [196, 204, 205]	EPR17341 C17F1	Abcam Cell Signaling
BRAF [115, 117]	VE1	SpringBio
MET [228, 231]	SP44	Ventana
PD-L1 [265, 266]	22C3 28-8 SP142 SP263 73-10	Dako Dako Ventana Ventana Dako

Individual tests, like series of immunohistochemistry, FISH, and PCR can lead to sample exhaustion, and thus, inconclusive, or false negative results in small biopsies and/or samples with low tumour cell counts by providing insufficient amounts of extracted nucleic acid [300]. Thus, biomarker testing practice is increasingly moving towards multigene technologies, such as the NGS [301].

Based on cost-effectiveness calculations, NGS is already preferable to single-gene tests when testing more than 4 targets simultaneously. In addition, this approach also realizes life-year gains, as calculated in several states [302, 303].

A not negligible aspect of predictive biomarker testing for NSCLC is turnaround time (TAT). Time consuming testing will result in delays in patient treatment, which may even fail due to patient deterioration. International recommendations suggest a TAT of 10 days from receipt of the sample to the communication of the result. The molecular test optimally should be performed in nearby laboratory. However, molecular testing is frequently centralized, which may prolong the TAT for logistical reasons (sample transport). Reflex testing also shortens the TAT, and supports optimal sample usage. In contrast, on-demand testing is more appropriate to avoid unnecessary tests, at the expense of the TAT [304]. It is important mentioning that the 10-day TAT recommendations is difficult to achieve with NGS in general. The sequencing approach is usually designed for 8 samples run simultaneously and the biochemistry is followed by a bioinformatic session of various complexity [304]. Any molecular techniques have their pros and cons laboratories should consider for their specific aims and needs. The choice of sequencing chemistry, device and software solutions also defines the acquisition of specific targets, the reagent requirements, the rate of the testing and turnaround times.

In lung cancer patients the tumour is frequently irresectable and/or the patient, or the tumor is unsuitable for bronchoscopic and/or transthoracic sampling. Small biopsy samples may often be not representative. In many of these cases, malignant pleural fluid is an alternative diagnostic specimen. MPE is present in about 15%–25% of lung tumours at diagnosis and occurs in 50%–60% of lung cancer patients overall during the disease [305–307]. However, the fluid cytology samples are sometimes also difficult to evaluate. It is recommended to prepare a cell block from the MPE specimen instead of the conventional cytospin smear. In general, cell blocks offer several advantages: the sample specimen can be examined as a tissue sample, it allows safe separation of activated mesothelial and tumour cells, and it provides accurate tumour typing. The cell block has higher diagnostic specificity and sensitivity than the cytospin smear, depending on tumor type (25, 53, 78% and 95% for squamous cell carcinoma, SCLC, adenocarcinoma and ovarian carcinoma, respectively [306, 308]. As a major benefit, cell blocks are suitable for immunohistochemical and molecular studies, including the investigation of all common predictive biomarkers for NSCLC therapy (EGFR, KRAS and ALK, ROS1, PD-L1 state).

## Conclusion

As described in our study, over the last 20 years, the increasing understanding of the molecular background of NSCLC has led to the identification of new therapeutic targets. Year after year, molecularly targeted treatment options are giving a growing group of patients with advanced NSCLC longer survival and better quality of life, without the life-threatening serious side effects of cytotoxic treatments. At the same time, patient selection has created unprecedented challenges for the diagnostic professions, particularly pathology departments. Further persistent work is needed to identify new molecular aberrations in addition to the current therapeutic targets, which will allow the use of more effective treatments for patients without the already identified driver mutation.
